# Exploring health system factors related to the quality of life of people with (a history of) cancer: a rapid review

**DOI:** 10.1186/s12955-026-02545-5

**Published:** 2026-05-13

**Authors:** Merel Engelaar, Iris van der Heide, Nanne Bos, Willemijn Schäfer, Carina Dantas, Laura Pinnavaia, Giovanni Apolone, Cinzia Brunelli, Augusto Caraceni, Norbert Couespel, Olatz Garin, Mogens Groenvold, Stein Kaasa, Gennaro Ciliberto, Claudio Lombardo, Ricardo Pietrobon, Gabriella Pravettoni, Aude Sirven, Hugo Vachon, Alexandra Gilbert, Jany Rademakers

**Affiliations:** 1 Netherlands Institute for Health Services Research (Nivel), Utrecht, The Netherlands; 2 Northwestern University, Chicago, USA; 3 SHINE 2Europe Lda, Coimbra, Portugal; 4 Department of Languages, Literatures, Cultures and Mediations, University of Milan, Milan, Italy; 5 Scientific Directorate, Fondazione IRCCS Istituto Nazionale dei Tumori, Milan, Italy; 6 Palliative Care, Pain Therapy and Rehabilitation Unit, Fondazione IRCCS Istituto Nazionale dei Tumori, Milan, Italy; 7 Department of Clinical Sciences and Community Health, Department of Excellence 2023-2027, University of Milan, Milan, Italy; 8 European Cancer Organisation, Brussels, Belgium; 9 Hospital del Mar Research Institute, Barcelona, Spain; 10 CIBER en Epidemiología y Salud Pública, CIBERESP, Madrid, Spain; 11 Bispebjerg/Frederiksberg Hospital and University of Copenhagen, Copenhagen, Denmark; 12 Oslo Universitetssykehus HF, Oslo, Norway; 13 IRCCS National Cancer Institute Regina Elena, Rome, Italy; 14 Organisation of European Cancer Institutes, Brussels, Belgium; 15 SporeData OÜ, Tallinn, Estonia; 16 Applied Research Division for Cognitive and Psychological Science, European Institute of Oncology IRCCS, Milan, Italy; 17 Department of Oncology and Haemato-Oncology, University of Milan, Milan, Italy; 18 Unicancer, Paris, France; 19 Quality of Life Department, European Organisation for Research and Treatment of Cancer, Brussels, Belgium; 20 Leeds Institute of Medical Research, University of Leeds, Leeds, UK; 21 Leeds Cancer Centre, St James’s University Hospital, Leeds, UK; 22 Department of Family Medicine, Care and Public Health Research Institute (CAPHRI), Maastricht University, Maastricht, The Netherlands

**Keywords:** Health system, Rapid review, Quality of life, Oncology, Europe

## Abstract

**Background:**

Cancer is a leading cause of death in Europe, and it has a major impact on the quality of life of those affected by it. Quality of life is a multifaceted concept affected by a range of factors, namely individual, organisational, and national health system factors. Despite existing research on individual and organisational aspects, little is known about the association between health system factors and quality of life. Therefore, the aim of this study is to explore the health system factors that relate to the quality of life of people with (a history of) cancer and to identify potential gaps in literature.

**Methods:**

We conducted a rapid review to gain insight into what is known in scientific literature regarding health system factors that are related to the quality of life of people with (a history of) cancer. We complemented our findings with a broad search in various grey literature databases.

**Results:**

The rapid review included 31 studies, which were supplemented by six health policy reports and one book chapter. Based on the review of scientific and grey literature, we constructed a list of ten health system factors that may relate to the quality of life of people with (a history of) cancer.

**Conclusions:**

We compiled a list of ten health system factors that may relate to the quality of life of people with (a history of) cancer. Seven factors were identified from and described in scientific literature. Three factors, namely ‘policy and vision’, ‘research and innovation’, and ‘quality of care delivery’, were identified in grey literature. The relation of these health system factors needs to be studied further to better understand what may impact on the quality of life of people with (a history of) cancer.

**Supplementary Information:**

The online version contains supplementary material available at 10.1186/s12955-026-02545-5.

## Background

Cancer is a leading cause of death and suffering in Europe, with 2.7 million newly diagnosed patients and 1.3 million deaths resulting from cancer in 2022 – accounting for over 20% of the total number of deaths in the European Union [1, 2]. Moreover, it has a major impact on the lives of those affected and their loved ones. Quality of life is increasingly recognised as an important outcome measure in clinical practice, since the burden of cancer and cancer treatment on patients is significant [3, 4]. Nonetheless, quality of life assessments are rarely implemented in standard oncological care. Similarly, quality of life is usually not considered as an aim or outcome measure in the development of policies and interventions within health systems and cancer control programs [5].

Quality of life is a multifaceted concept, that can be affected by the interaction between individual, organisational, and system-level factors. Individual or micro-level factors include, for example, mental resilience and the social support received from close relatives. There is a large body of evidence that demonstrates the associations between these individual factors and the quality of life of people with (a history of) cancer [6–9]. Additionally, several studies have revealed the associations between organisational or meso-level factors and the quality of life of people with (a history of) cancer, for instance, the experienced quality of the care received [10, 11]. However, less is known about the association between national health system or macro-level factors and the quality of life of people with (a history of) cancer.

The World Health Organization (WHO) defines a health system to include “all the activities whose primary purpose is to promote, restore or maintain health” [12]. It highlights four functions as a basis to better understand and evaluate health systems and their performance. Firstly, *governance* ensures that strategic policy frameworks exist and are combined with effective oversight, coalition-building, and accountability. Secondly, *resource generation* warrants that a health system has all the inputs it needs to function, including health workers, medical equipment, and pharmaceuticals. Thirdly, *financing* constitutes an integral function of a health system: raising and spending money on health care. Lastly, *service delivery* encompasses all inputs into care processes taking place in an organisational setting and leading to the delivery of health services. The WHO recognises that these health system functions can be related to health system outcomes in many ways [13].

International differences in health care delivery, quality and outcomes suggest substantial variabilities in the performance of national health systems that care for and treat people with (a history of) cancer [5, 14]. Hence, health system factors are expected to influence the quality of life of people with (a history of) cancer, as well as interact with individual and organisational factors. A deeper understanding of factors contributing to differences in quality of life across countries may be obtained by gaining insight into the national health system factors that are associated with the quality of life of people with (a history of) cancer. Policies, strategies, and interventions that aim to enhance patients’ quality of life may be guided by this insight as well.

This study aims to explore the health system factors that relate to the quality of life of people with (a history of) cancer and to identify the potential gaps in the literature on this topic. The following research questions will be addressed:

1. What health system factors have been identified in scientific literature in relation to the quality of life of people with (a history of) cancer?
2. What health system factors are *potentially* related to the quality of life of people with (a history of) cancer, based on grey literature?

## Methods

### The EUonQoL project & Patient and Public Involvement

This study was conducted as part of the research project called “Quality of Life in Oncology: measuring what matters for cancer patients and survivors in Europe” (EUonQoL). The EUonQoL project aims to review existing quality of life scales in order to develop new metrics by harnessing the strengths and overcoming the limitations of previous tools. The EUonQoL-Kit, a new set of quality of life questionnaires designed for patients under active treatment, survivors, and patients in need of palliative care, will be the product of this effort. It will form a new digital system for quality of life self-assessment, available in several European languages and developed from the patient perspective [5]. The overall project is based on Patient and Public Involvement (PPI) research principles, by involving persons with lived experience of cancer as ‘co-researchers’ throughout all research stages [15].

Co-researchers also played a meaningful role in this study. Their contributions included: (a) suggesting to consult grey literature in addition to scientific literature; (b) helping with the interpretation of the overall results; (c) co-authoring this article. They were not involved in conducting the rapid review, as they had not been introduced to the project at that time due to the tight timeline of the EUonQoL project.

### Theoretical framework

**Fig. 1:**
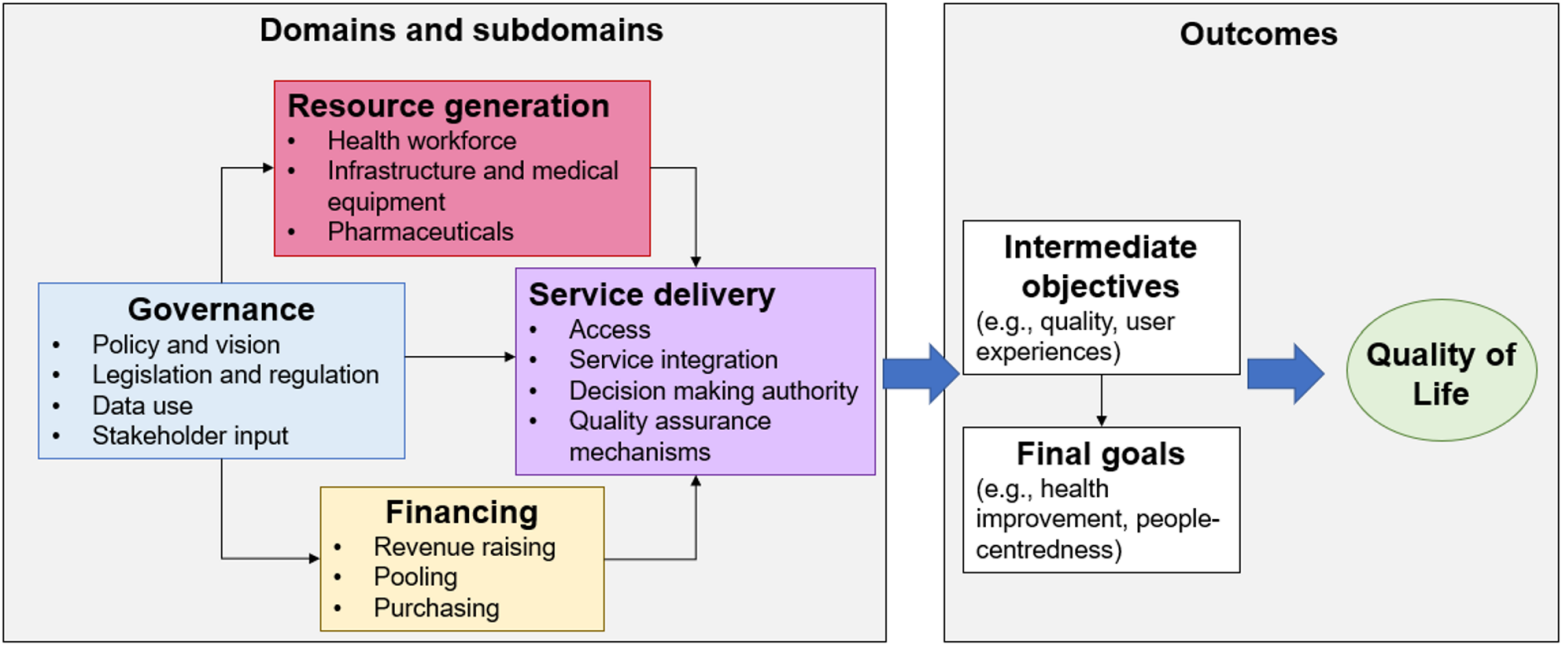
The Health System Performance Assessment framework, adapted from Papanicolas et al. [13]

We selected the WHO’s Health System Performance Assessment framework [13] as the foundational model for our research. This model was slightly adapted and simplified by two researchers (ME and WS), tailoring it to our specific objective of determining features of health systems that may be associated with the quality of life of people with (a history of) cancer (Fig. 1). We adapted the framework by adding the factor ‘access’ to the service delivery domain, as several aspects of access can be viewed as systemic aspects of service delivery. This was different from the original framework, which considers access as an objective or outcome. Furthermore, we simplified the model by limiting the descriptions of intermediate objectives and final goals, which are not the focus of the current study. Lastly, we added quality of life as an outcome because the quality of life of patients is expected to be affected by health system factors.

### Rapid review

#### Study design

We conducted a literature review on the association between health system factors and the quality of life of people with (a history of) cancer. We opted for a rapid review, a method that streamlines traditional systematic review methods to synthesise evidence within a shortened timeframe [16]. This method was chosen because the research is exploratory, and a full systematic review was deemed premature at this stage. Additionally, at this point we were looking for relevant factors in breadth rather than in depth. Lastly, this method was chosen to accommodate the tight timeline of the EUonQoL project. As there are still no reporting guidelines available for rapid reviews, we used the Preferred Reporting Items for Systematic reviews and Meta-Analyses (PRISMA) 2020 checklist [17].

#### Data source and search strategy

In May 2023, we performed a rapid review in PubMed to gain insight into what is already known about the health system factors related to the quality of life of people with (a history of) cancer. The search strategy for the rapid review (Table 1) was derived from an existing search string used for another (related) systematic review within the EUonQoL project [6]. The search string was refined based on an exploration of existing keywords and search words, further discussed multiple times, and agreed upon by two researchers (ME and WS).

**Table 1:** Search strategy (PubMed)

1.	“health system*“[Title/Abstract] OR “health care system*“[Title/Abstract] OR “healthcare system*“[Title/Abstract] OR “health economic*“[Title/Abstract] OR “health care economic*“[Title/Abstract] OR “healthcare economic*“[Title/Abstract] OR “health cost*“[Title/Abstract] OR “health care cost*“[Title/Abstract] OR “healthcare cost*“[Title/Abstract] OR “health expenditure*“[Title/Abstract] OR “health care expenditure*“[Title/Abstract] OR “healthcare expenditure*“[Title/Abstract] OR “health policy“[Title/Abstract] OR “health care policy“[Title/Abstract] OR “healthcare policy“[Title/Abstract] OR “health policies“[Title/Abstract] OR “health care policies“[Title/Abstract] OR “healthcare policies“[Title/Abstract] OR “medical education*“[Title/Abstract] OR “nursing education*“[Title/Abstract] OR “health workforce“[Title/Abstract]
2.	factor*[Title/Abstract] OR indicator*[Title/Abstract] OR characteristic*[Title/Abstract] OR predictor*[Title/Abstract] OR determinant*[Title/Abstract]
3.	affect[Title/Abstract] OR effect[Title/Abstract] OR related[Title/Abstract] OR impact[Title/Abstract]
4.	“quality of life“[Title/Abstract] OR qol[Title/Abstract] OR “health-related quality of life“[Title/Abstract] OR hrqol[Title/Abstract]
5.	cancer[Title/Abstract] OR tumor[Title/Abstract] OR tumour[Title/Abstract] OR neoplasm[Title/Abstract] OR carcinoma[Title/Abstract] OR oncolog*[Title/Abstract]
6.	#1 AND #2 AND #3 AND #4 AND #5

#### Inclusion and exclusion criteria

Articles were deemed eligible for inclusion if the research subjects were adults with (a history of) cancer; if quality of life was measured; and if health system factors were studied as an explanatory variable of quality of life. Protocol papers were excluded from our study. All types of studies were included in our review (e.g., randomised controlled trials, cross-sectional studies, review studies). No limit was set to publication dates.

#### Study selection

The study selection process was performed using Rayyan [18]. Search results were imported into Rayyan, and duplicates were identified and removed. Two researchers (ME and WS) screened titles and abstracts. After the initial screening, the researchers discussed their results, resolved conflicting inclusion or exclusion decisions, and refined the inclusion and exclusion criteria. One researcher (ME) screened again all included articles to confirm that they complied with the refined inclusion and exclusion criteria. Full-text screening was performed by one researcher (ME), and any uncertainties regarding final inclusion were resolved through discussion with a second researcher (WS). Articles that were not written in English, were translated using DeepL Translator prior to full-text screening.

#### Data extraction

The articles that were included for data extraction were all read in full text by one researcher (ME), and the relevant data were extracted using a pre-defined data extraction form. The variables that were collected using this data extraction form included: author(s), publication year, study type, country, study population, health system domain studied, health system factor studied, outcome measure(s) related to quality of life and results on quality of life.

#### Assessment of methodological quality

The methodological quality of the included studies was not assessed to facilitate the rapid, exploratory nature of our research.

### Grey literature search

The rapid review generated limited results, and therefore, it was suggested by our co-researchers to broaden our literature review by including grey literature resources too. Thus, in addition to our review of scientific literature, we also searched for health policy reports on Google Scholar and websites such as those of the Organisation for Economic Cooperation and Development (OECD) and the WHO. It is important to note that the factors described in the grey literature are *potentially* related to quality of life, as their association has not been scientifically studied yet.

## Results

### Rapid review

#### Results of the selection process

**Fig. 2:**
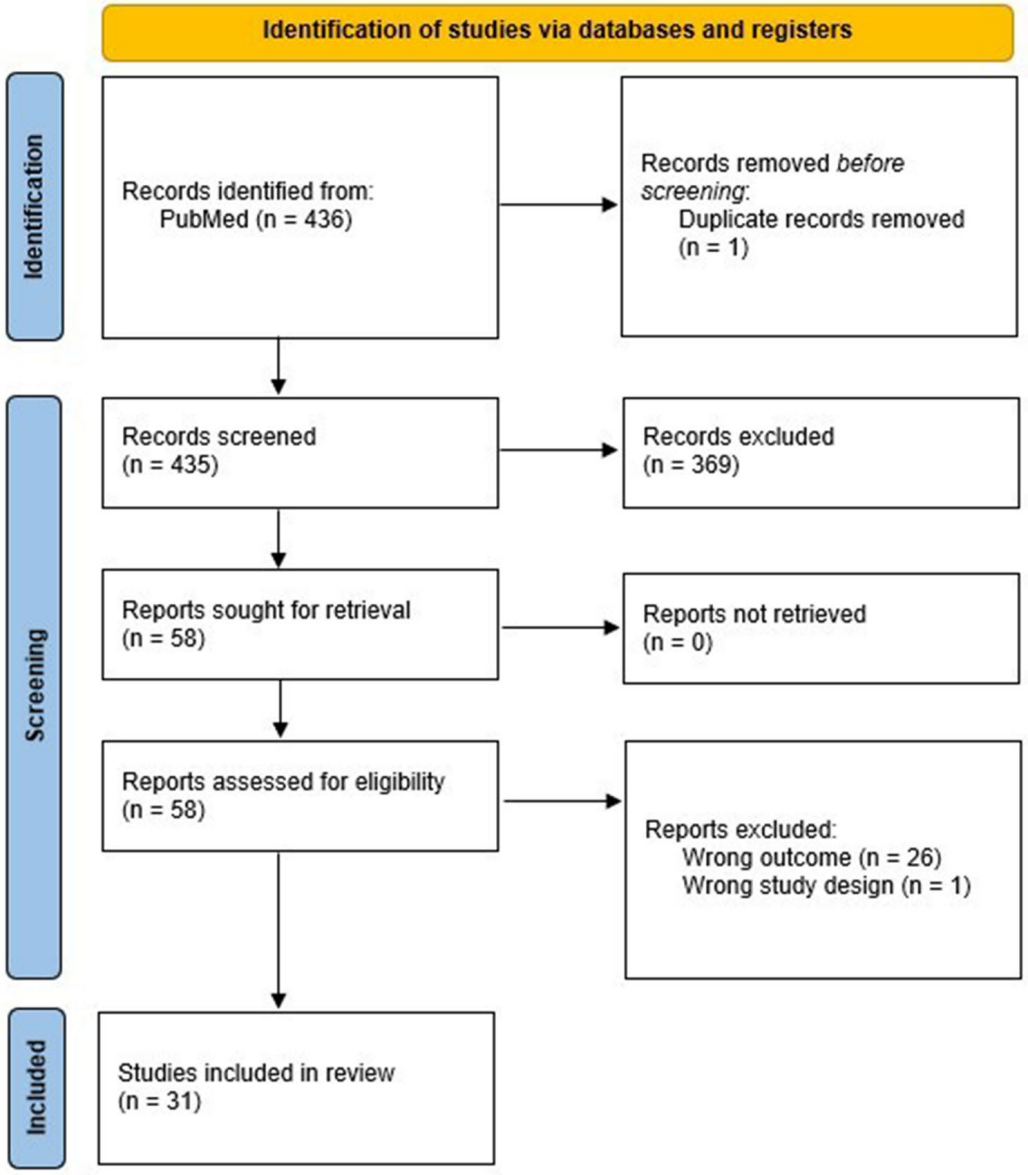
PRISMA 2020 flow diagram of the study selection process

Our search in PubMed identified 436 references. After removing a duplicate, 435 records remained for title and abstract screening. A total of 58 articles were included for full-text screening, out of which 27 were excluded for various reasons: most reports (*n* = 26) did not describe health system factors as an explanatory variable of quality of life, and one report described a pilot study for a new quality of life instrument. This resulted in 31 studies that were included in our review. The selection process is shown in the flow diagram in Fig. 2.

#### Characteristics of the included studies

Almost all included articles have been published in the last fifteen years, except for one study, which was published in 2000. About one-third of the selected articles were reviews; the remaining studies were original articles, including cross-sectional studies and randomised controlled trials. Of these original studies, only four were performed in Europe (the Netherlands, Ireland, and the United Kingdom) while the majority were performed in the United States and Asia. An overview of study characteristics can be found in Supplementary material 1. The domain reported in most articles was financing (*n* = 15), followed by service delivery (*n* = 14). One article reported on the resource generation domain, and one article addressed the governance domain.

#### Financing factors related to quality of life

Fifteen articles reported on the financing domain in relation to the quality of life of people with (a history of) cancer. In general, it was stated that persons who experience financial toxicity (including financial hardship, financial worry, financial burden, financial difficulty) have worse quality of life outcomes on the physical, functional, and psychosocial domains [19–29]. Studies also reported more specific financial characteristics, all of which have a significant negative influence on an individuals’ quality of life: financial consequences of being retired, not having paid work, not being able to do paid work, low/reduced household income, not having health insurance, increased out-of-pocket costs, avoiding care because of costs, and health care costs exceeding annual household income [20, 21, 24, 28, 30, 31]. While these factors are measured individually, a direct underlying factor is the financing of the health system. It was also reported that the total per capita health expenditures factor significantly affected quality of life outcomes, with patients from countries with low per capita health expenditures having significantly lower levels of quality of life [32, 33].

#### Service delivery factors related to quality of life

Fourteen articles identified reported on the service delivery domain. General aspects of service delivery that were found to be associated with quality of life included patient-centred communication, patient involvement in care, provision of information, and health care provider support [34–36]. Specific aspects of service delivery that were described as having a positive association with quality of life outcomes included having a cancer care coordinator and early integration of palliative care in a community care setting [37, 38]. The studies that reported on access to service delivery described that unmet supportive care needs in domains such as physical, psychological, and health system and information needs, negatively affected quality of life outcomes [39–43]. In four studies, some aspects of service delivery were *not* shown to have an effect on quality of life outcomes: advanced practice nursing interventions, nurse-led follow-up at home, weekly symptom telemonitoring with automated reporting of problematic symptoms to the clinical team, and telerehabilitation [44–47]. As a potential explanation, it would appear that it may not be realistic to presume that at an advanced stage of the disease quality of life would be significantly influenced by a regular visit from a nurse [45]. Additionally, it is argued that the effectiveness of telehealth programs may be context- and outcome-dependent, for instance, patients may experience barriers due to the lack of physical interaction and inexperience in using technology [46, 47].

#### Resource generation factors related to quality of life

In the article reporting on resource generation in relation to the quality of life of people with (a history of) cancer, the influence of hospital and surgeon volume on outcomes following oesophageal cancer surgery was evaluated. The authors describe a non-significantly improved quality of life among patients operated on at high-volume hospitals compared with low-volume hospitals. The authors suggest that centralisation’s association with reduced postoperative mortality may also be linked to improvements in quality of life [48].

#### Governance factors related to quality of life

In the article reporting on governance factors, the quality of life of women with breast cancer in Colombia is described. The authors report a significantly higher overall quality of life among women enrolled in the contributory health insurance system. This system – which provides comprehensive health care at the cost of a mandatory co-payment – leads to inequitable access to cancer treatment and care, as those who are not enrolled in the system face greater barriers, with implications for their quality of life [49].

### Grey literature search

In our grey literature search we found six health policy reports and one book chapter. An overview of the publication characteristics can be found in Supplementary material 2. Most information was found regarding the service delivery domain, in addition to findings on the domains of resource generation and governance. However, we did not find any reports on the financing domain.

#### Service delivery factors related to quality of life

Regarding service delivery, one report described the potential added value of digital tools for patient monitoring, as it can save costs associated with unnecessary visits and delayed interventions, while improving quality of life [50]. Other reports described the positive effects associated with service provision by multidisciplinary teams on the quality of life of people with (a history of) cancer [51, 52]. Furthermore, the potential benefits of screening and early diagnosis [53, 54], as well as the provision of supportive care and the existence of quality assurance systems [52], have been described in terms of the quality of life of people with (a history of) cancer.

#### Resource generation factors related to quality of life

As to resource generation, it was described that lack of systematic dissemination and implementation of knowledge and innovation in daily practice can potentially hinder improvements in quality of life [51, 55]. Additionally, it was described that referral to centralised points of expertise may negatively affect the quality of life, due to increased travel distances and costs for patients and their families [52].

#### Governance factors related to quality of life

In the governance domain, authors of one report hypothesised that an integrated national approach, such as National Cancer Control Programmes, is of importance for the quality of life of people with (a history of) cancer [51]. Moreover, it was reported that policies ensuring fair access to services, employment, and insurance may especially be important for cancer survivors [56].

#### Summary of health system factors (potentially) related to quality of life

Based on our exploration of scientific and grey literature, we have summarised the health system factors that were found to be (potentially) related to the quality of life of people with (a history of) cancer (Table 2). It is important to distinguish between scientific and grey literature: the factors described in the grey literature are *potentially* related to quality of life, as their association has not been scientifically studied yet. The comprehensive list includes ten factors: two related to the governance domain, two related to resource generation, one related to the financing domain and five related to service delivery.

**Table 2:** Overview of health system factors (potentially) related to the quality of life of people with (a history of) cancer

Health system domains	Factors / subdomains	Literature source	Findings
Governance	Policy and vision	Grey literature	• National Cancer Control Programmes are increasingly seen as essential to improving the quality of life for people with (a history of) cancer [51]
	Legislation and regulation	Scientific and grey literature	• Being enrolled in a contributory health insurance system positively affects quality of life, however, it also leads to inequitable access to cancer treatment and care [49] • It is advisable to improve the quality of life of cancer survivors through policies to ensure their access to psychosocial services, fair employment practices, and health insurance [56]
Resource generation	Workforce capacity and training	Scientific and grey literature	• Quality of life improves non-significantly among patients operated on at High-Volume Hospitals compared with Low-Volume Hospitals [48] • Health migration generated by centralised referral should be limited, as it implies an adverse impact on the quality of life of patients [52]
	Research and innovation	Grey literature	• Incorporating innovations in the care continuum may also positively affect the quality of life of people with (a history of) cancer and all the populations at risk [51] • Recent developments in cancer diagnosis and treatment have dramatically improved the quality of life for people with (a history of) cancer [55]
Financing	Affordability of treatment	Scientific literature	• Patients from countries with low per capita health expenditures have lower levels of quality of life [33] • The total per capita health expenditures predictor has a statistically significant impact on quality of life [32] • Increased out-of-pocket costs impact quality of life negatively [31] • High out-of-pocket costs impact quality of life negatively [24]
Service delivery	Accessibility and timeliness of care	Scientific and grey literature	• Some studies show that outpatient or home palliative care improve quality of life [38] • Digital tools for patient monitoring may improve quality of life [50]
	Availability of care and support services	Scientific and grey literature	• Unmet supportive care needs negatively impact quality of life [39, 40, 42, 43] • Healthcare provider support positively impacts quality of life [36] • Psychosocial interventions have been demonstrated to be effective in improving psychosocial outcomes in people with (a history of) cancer, including quality of life [52]
	Integration, coordination, and continuity of care	Scientific and grey literature	• Having a cancer care coordinator has a non-significant positive impact on quality of life [37] • Multidisciplinary teams result in better clinical and process outcomes for people with (a history of) cancer in terms of quality of life [51] • Specialist cancer nursing has been associated with improved quality of life outcomes [52] • Organisation of cancer care by multidisciplinary teams is key to the provision of high-quality cancer care to patients and people with (a history of) cancer’ quality of life across Europe [52]
	Quality of care delivery	Grey literature	• Early diagnosis of cancer leads to a better quality of life [53] • The benefits of early palliative care intervention have also been reported in terms of quality of life [52] • The quality of life of patients may improve when cancer is diagnosed at an early stage [54] • The implementation of quality assurance systems allows for the protection and enhancement of patient quality of life [52]
	Patient-centredness	Scientific literature	• Patient-centred communication positively impacts quality of life [34] • Patient-centred communication and patient involvement positively impact quality of life [35]

## Discussion

This study aimed to explore the health system factors that relate to the quality of life of people with (a history of) cancer and to identify the potential gaps in the literature on this topic. Based on our review of scientific and grey literature, we constructed a list of ten health system factors, covering the four health system domains. The governance factor ‘legislation and regulation’, the resource generation factor ‘workforce capacity and training’, the financing factor ‘affordability of treatment’, and the service delivery factors ‘accessibility and timeliness of care’, ‘availability of care and support services’, ‘integration, coordination, and continuity of care’ and ‘patient-centredness’, were all found in the scientific literature to relate to the quality of life of people with (a history of) cancer. Additionally, the governance factor ‘policy and vision’, the resource generation factor ‘research and innovation’, and the service delivery factor ‘quality of care delivery’, were hypothesised as relating to quality of life within grey literature resources.

In our exploration of scientific and grey literature, we found a varying number of resources across health system domains. This is especially the case for the financing and governance domains. In the scientific literature, financing receives considerable attention; however, this is not mirrored by the grey literature, where we did not find reports on financing. This may be a direct result of our search strategy for the rapid review, which features several search terms related to financing, as opposed to fewer search terms related to the other health system domains. The search strategy was developed in an iterative process based on an exploration of commonly used keywords and search words, thereby, in hindsight, limiting our focus to topics that are frequently reported on. Additionally, or even as a result of our search strategy, many of the scientific papers originated from the United States, while most of the grey literature originated from Europe. Hypothetically, the fact that healthcare access is universal in Europe, but a considerable proportion of Americans remain uninsured [57], leads to an enhanced focus on financial aspects in the United States resources. For the governance domain, few resources were found in scientific and grey literature. We can argue that governance creates the preconditions for all the other factors, as illustrated in both our adapted framework (Fig. 1) and the original Health System Performance Assessment framework [13]. Speculatively, governance relates to the quality of life of people with (a history of) cancer by influencing the other domains and therefore does not directly appear in the literature as a relevant factor.

For future research, there is undoubtedly a need to explore further the extent to which the various health system factors relate to the quality of life of people with (a history of) cancer. This is especially true for the governance domain, for which little evidence was found in the literature. For example, it could be studied whether the quality of life of people with (a history of) cancer changes after the initiation of a National Cancer Control Programme. Further research could also substantiate the vital role that service delivery may play, as suggested by both scientific and grey literature. For instance, this could be carried out through a structured national/regional data collection plan on the identified health system factors, thereby enabling cross-country comparisons. In line with this, we wish to highlight the potential of the EUonQoL-Kit here, given that the availability of a new, valid and reliable questionnaire could be used on both a national and a European level to verify the impact of health systems on quality of life, as well as to identify all the different factors that potentially influence it [58].

To our knowledge, this study is the first to explore the relationship between health system factors and the quality of life of people with (a history of) cancer. In doing so, we used various sources of information, including scientific and grey literature, to ensure a comprehensive examination of available resources. The insights we present here, serve as a starting point for future research, as well as to inform policymaking related to cancer care at the system level.

This study has several limitations. Firstly, it has focused on health system factors, while interactions with individual and organisational factors related to cancer prevention, treatment, and care are also occurring. Additionally, socioeconomic and demographic characteristics of populations within countries are also likely to play a role in explaining differences in the quality of life people with (a history of) cancer have within or between countries. Secondly, we recognise that the term ‘quality of life’ can have varying definitions and interpretations, depending on the context in which it is used. As the current study is exploratory, we have not made a distinction between the different definitions and measurement possibilities of quality of life. Thirdly, the impact of health system factors on quality of life might differ depending on the study population (e.g., persons undergoing treatment or those who are in remission, breast cancer patients or colorectal cancer patients). We have not distinguished between disease phases and cancer types in this study. For future research, a robust strategy should (1) broaden explanatory domains beyond health-system factors to include population-level socioeconomic and demographic characteristics; (2) explicitly define and harmonise quality of life definitions and measurements; and (3) acknowledge heterogeneity across study populations by stratification and interaction testing. For example, the interactions between health system factors and socioeconomic/demographic characteristics could be systematically examined to identify subgroups for which health system improvements yield the greatest quality of life gains.

With regard to methodological limitations, our search strategy did not give equal weight to all health system domains, thereby leading to results that privilege some aspects more than others. A broader, more extensive search strategy would potentially have yielded more results in our rapid review, leading to more robust and balanced findings.

## Conclusions

In conclusion, within this exploratory study we constructed a list of ten health system factors that may relate to the quality of life of people with (a history of) cancer. Seven of these factors were described in scientific literature as being related to the quality of life of people with (a history of) cancer. Three factors were hypothesised as relating to quality of life within grey literature resources and require further study. This explorative study serves as a starting point for future research, which, given the limited evidence identified, is highly recommended. These future findings will offer the potential to strengthen and improve policies and strategies related to cancer care at the system level.

## Electronic Supplementary Material

Below is the link to the electronic supplementary material.

Supplementary Material 1

Supplementary Material 2

## Data Availability

The data that support the findings of this study are available from the corresponding author upon reasonable request.

## Abbreviations

WHO: World Health Organisation
EUonQoL: “Quality of Life in Oncology: measuring what matters for cancer patients and survivors in Europe”
PPI: Patient and Public Involvement
OECD: Organisation for Economic Cooperation and Development
