# Supplementary material for: Exploring health system factors related to the quality of life of people with (a history of) cancer: a rapid review
Source: Health Qual Life Outcomes. 2026 May 13;24:90. doi: 10.1186/s12955-026-02545-5 (PMC13340385; doi:10.1186/s12955-026-02545-5)
Supplement: Supplementary file 1 — Supplementary Material 1 [file 12955_2026_2545_MOESM1_ESM.docx]

**Supplementary material 2: Grey literature publication characteristics**

| **Author(s)** | **Publication year** | **Publication type** | **Country** |
| --- | --- | --- | --- |
| Organisation for Economic Co-operation and Development (OECD) | 2024 | Report | Europe |
| European Commission | 2021 | Report | Europe |
| European Observatory on Health Systems and Policies | 2021 | Report | Europe |
| Leemrijse et al. | 2021 | Report | Europe |
| European Parliament | 2020 | Report | Europe |
| National Institute of Public Health of the Republic of Slovenia & European Observatory on Health Systems and Policies | 2013 | Report | Europe |
| Institute of Medicine and National Research Council | 2005 | Book | United States |
