# Supplementary material for: Exploring health system factors related to the quality of life of people with (a history of) cancer: a rapid review
Source: Health Qual Life Outcomes. 2026 May 13;24:90. doi: 10.1186/s12955-026-02545-5 (PMC13340385; doi:10.1186/s12955-026-02545-5)
Supplement: Supplementary file 2 — Supplementary Material 2 [file 12955_2026_2545_MOESM2_ESM.docx]

**Supplementary material 1: Rapid review study characteristics**

| **Author(s)** | **Publication year** | **Study type** | **Country** | **Study population** |
| --- | --- | --- | --- | --- |
| Liang et al. | 2023 | Review | N/A | Patients with gynaecological cancer |
| Burse et al. | 2022 | National telephone survey | United States | Cancer survivors |
| Jiang et al. | 2022 | Review | N/A | Cancer survivors |
| Lee et al. | 2022 | Review | N/A | People with chronic diseases (including cancer) |
| Sritan | 2022 | Review | Thailand | Patients with colorectal cancer with a stoma |
| Fitch & Longo | 2021 | Review | Canada | Cancer patients and survivors |
| Udayakumar et al. | 2021 | Review | Low- and middle-income countries | Cancer patients |
| de la Cruz & Delgado-Guay | 2021 | Review | N/A | Patients with advanced cancer |
| Nedjat-Haiem et al. | 2021 | Cross-sectional study | United States | Older Latinos with chronic diseases (including cancer) |
| Pozzar et al. | 2021 | Cross-sectional descriptive survey | United States | Patients with ovarian cancer |
| Del Vecchio et al. | 2021 | Population-based survey | United States | Cancer survivors |
| Dunn et al. | 2021 | Cross-sectional study | Australia | Cancer survivors |
| Drury et al. | 2020 | Descriptive qualitative study | Ireland | Colorectal cancer survivors |
| Jie et al. | 2020 | Cross-sectional study | China | Acute leukaemia patients |
| Mady et al. | 2019 | Cross-sectional survey | United States | Head and neck cancer survivors |
| Ting et al. | 2019 | Cross-sectional survey | Malaysia | Urologic cancer patients |
| Sodergren et al. | 2019 | Multicentre prospective cohort study | United Kingdom | Patients with colorectal cancer |
| Chen et al. | 2018 | Survey | China | Lung cancer patients |
| Casilla-Lennon et al. | 2018 | Cross-sectional study | United States | Bladder cancer patients |
| Jansen et al. | 2017 | Cross-sectional study | The Netherlands | Head and neck cancer patients treated with total laryngectomy |
| Kimman et al. | 2015 | Cross-sectional survey | Southeast Asia | Newly diagnosed cancer patients |
| Zafar et al. | 2015 | Prospective cohort study | United States | Cancer survivors |
| Davis et al. | 2015 | Review | N/A | Patients with serious illnesses  (including cancer) |
| Yount et al. | 2014 | Randomised controlled trial | United States | Advanced lung cancer patients |
| Uitdehaag et al. | 2014 | Randomised study | The Netherlands | Patients with incurable upper gastrointestinal cancer |
| Popovic et al. | 2013 | Review | N/A | Patients with advanced cancer |
| Popovic et al. | 2013 | Review | N/A | Patients with early stage cancer |
| Park & Hwang | 2012 | Questionnaires | Korea | Women with recurrent breast cancer |
| Rouvelas & Lagergren | 2010 | Review | N/A | Oesophageal cancer patients |
| Zapata & Romero | 2010 | Cross-sectional study | Colombia | Women with breast cancer |
| Ritz et al. | 2000 | Randomised clinical trial | United States | Women with breast cancer |
